# Supplementary material for: PISA data clusters reveal student and school inequality that affects results
Source: PLoS One. 2022 May 11;17(5):e0267040. doi: 10.1371/journal.pone.0267040 (PMC9094565; doi:10.1371/journal.pone.0267040)
Supplement: Attachment — Submitted filename: Response to Reviewers.pdf [file pone.0267040.s001.pdf]

Rebuttal letter

Response to the Academic Editor

Thank you very much for taking the time to consider my manuscript. It combines seemingly disparate fields and I appreciate the effort to seek out reviewers from different areas of research, and I support your intention to seek out also a third reviewer.

Below follow my comments and corresponding changes. I must mention though that it is my impression that most of the remarks made by reviewer 1 are a result of misunderstandings and/or lack of insight into statistical methods beyond those that are standard in the social sciences. I have however tried to improve the manuscript as requested.

Kind regards,
Magnus Neuman

ACADEMIC EDITOR: […] To meet Plos criteria for publication, the author must, at minimum, address the concerns of reviewer one related to the measurement of PISA scores (Reviewer 1's point 2) and better ground the study in the relevant literature and theory. The statistical approach is novel in this field of study; the author just needs to make clear what new knowledge this approach affords over standard statistical approaches used in previous studies or how this study replicates, in a conceptual, previous results.

This relates to point 12 of reviewer 1 and is partly addressed in my response to that. I have added another two references [16, 17] that contain results on the relation between educational outcome and socio-economic status. However, previous work offer few nuances beyond stating that academic results improve with increasing socio-economic status (as stated in the introduction), and I believe that the present work can fill that gap.

The main results of the present study (as stated in the abstract, results and discussion) are the identification of a cluster of countries characterized by high performance and less privileged students, together with variations between public and private schools, and the novel approach to studying these data. These results are novel and should be of general interest.

The second sentence of the discussion has been changed in order to further emphasize the contrast between previous work and the present work.

Furthermore, I suggest changing the title to ”PISA data clusters reveal student and school inequality that affects results”, in this way narrowing down the scope of the title and pointing more explicitly in the direction of the novel results.

Response to Reviewers

Reviewer #1

Thank you very much for your valuable comments. I have placed my clarifications and declared the corresponding changes to the manuscript after each of your points below.

I have the following comments on the paper "The PISA data reveal student and school inequality that affect results."
1. The Methods section should be presented before the Results and Discussion sections.

It is commonplace in many journals today, including PLOS ONE, to place the methods section last. This improves readability.

2. The authors calculated students' "normalized points" Pi by dividing points scored per question by the difficulty of each question. The nominator is the difference between the max difficulty and the min difficulty of each student (page 8). First, however, the max and min difficulties of questions answered vary across students, and the gap between the max and min difficulty also varies across students. Therefore, using the nominator as the gap of max and min difficulty of individual students does not normalize PISA scores across the student distribution. Second, PISA test scores have been weighted to take into account the difficulty of different questions and allow meaningful comparisons across students/cohorts/countries. PISA test score is a better measure of student performance than Pi calculated by the authors. Also, the construction of Pi should be placed in the Methods section.

I found two typos in this section and apparently I have also expressed myself unclear. I would like to thank the reviewer for drawing my attention to this. The first remark made by the reviewer is a result of a misunderstanding. The max and min difficulties refer to globally calculated difficulties, including all items/questions for all students, as reported in the OECD technical report. This is therefore a proper normalization of the item difficulties. I have rewritten this sentence and hope that this will avoid similar misunderstandings.

I also found a typo in Eq. 1, where one of the indices was missing, which might have caused confusion. This is now corrected.

In the second remark, the reviewer claims that ”PISA test scores” are a better measure of student performance than the one proposed in the article. Firstly, there is no such thing as ”PISA test scores” on a student level. Secondly, assuming that the reviewer alludes to ”plausible values”, which are reported at student level, there are two arguments against using these: 1. They should not be used to calculate student level results, and 2. This would be significantly less transparent than using the raw data as in the present work. I changed the sentence following Eq. 1 to emphasize this more.

Important parts of the methods are often placed in the Results section. Once again, this is to improve readability.

3. How do the authors define "privileged students" using the ESCS score? What is the cut-off point, and why is that point chosen as a cut-off?

The division of students into groups/clusters is a result of the network representation of the PISA data and the clustering of the network with state-of-the-art methods including Infomap. No claim has been made about the existence of a strict cut-off, and I see no reason to clarify this further. Continuing the analogy with climate regions, what is the cut-off (in terms of eg. precipitation) between tropical and sub-tropical regions?

4. Why did the authors use bootstraps (page 7) to calculate summary statistics? The PISA sample is supposed to be representative at the country level. They can use any statistical software to calculate the presented results. From what I read here, the results are the mean of test scores and the correlation between test scores and ESCS for different groups of students.

The bootstrapping is prescribed by the OECD, as described in the methods section. The first sentence in ”Significance analysis” is now changed in order to make this clearer.

5. "Significance analysis" (page 7): It's not clear what the purpose of the significance analysis presented in this section is. If I understand correctly, the authors use significance analysis to test for the difference between mean scores across students from different economic backgrounds and/or whether the correlation coefficient between test scores and ESCS is statistically significant. If so, t-test and regressions are methods commonly used in social science for these purposes. If the authors decide to use modules and module node assignments, please cite a peer-reviewed published paper that has used this method to investigate similar issues in education or, in addition, present t-tests and regressions to show that the two approaches are similar.

T-tests and regressions are not applicable when working with networks and module node assignments. Instead, significance analyses involving bootstrapping are often used. This is straightforward in fields such as network science, and the reference provided (24, in Phys Rev. E) should suffice. The method we use has the advantage of using the relatively transparent Jaccard index (based on set theory) instead of eg. mutual information (based on entropy).

6. "Building the network of countries and space nodes" (page 9): What is the purpose of using this method? What do the authors mean by "where the country has a larger fraction of its students than the average of all countries"? Do they mean a larger fraction of "privileged students" in the whole student population? If so, the statistic they need to calculate here is the deviation from the mean of the fraction of "privileged students", and that can be calculated using any statistical software.

The purpose of using this method, as stated in the introduction, is that we can benefit from the development of methods in network theory, which have shown useful in eg. theoretical ecology to find large scale patterns in data. The other remarks made by the reviewer are somewhat unclear, but I do believe that the method is sufficiently explained in one figure, the introduction and in the results and methods sections.

7. Page 9: What is "Figure ??"

This is a LaTeX related typo that I now have corrected.

8. The authors need to provide the take-away point from each figure presented in the results section.

I believe that this is already in place.

9. Figure 1 looks like the authors map PISA's deciles with ESCS scores, but I'm unsure.

I do not understand this remark, but I have added a short explanation of Fig. 1a to the figure caption.

10. The authors also conclude that students from privileged backgrounds performing better in Sweden results from school segregation. However, at least in the US, private schools are likely to select students with involved parents, and families with involved parents are likely to have higher test results regardless of ESCS status. Also, to attract students, private schools may provide more educational resources to students and have better teachers than public schools. Can you control for these different effects?

This is a very interesting topic, but unfortunately, I believe the present dataset does not contain enough information to answer these questions.

11. Cultures, such as China, where acceptance into top universities is dependent on doing well on exams, means that Chinese students in Public schools are also likely to attend after-school private schools. Is there a way that you can control for students who attend both public schools and private schools?

I believe this information is not included in the present dataset.

12. The authors' main conclusions are 1) students with a higher socio-economic status (SES) perform better than students with a lower SES, and 2) the extent of the performance gap varies across countries. The authors should reference more of the literature on higher SES students performing better than low SES students, which is well established in the social science literature. They should also reference the literature on human capital theories that provide explanations for the difference in academic achievement between students from different economic backgrounds.

I already reference previous research on this issue, but I have now added further references to previous research [16, 17]. The main conclusion is however the identification of a cluster of countries characterized by high performance and less privileged students, together with variations between public and private schools, and the novel approach to studying these data. This is clearly stated at several locations in the manuscript, including the abstract, but to further emphasize this I suggest changing the title to ”PISA data clusters reveal student and school inequality that affects results”.

Being a multidisciplinary study, the list of referenced literature cannot be exhaustive from the point of view of every related field. One example of this is that I do not relate the findings to human capital theories, and neither do most of the other papers that deal with connections between educational outcome and socio-economical status.

Reviewer #2:

Thank you very much for the time invested and for the valuable comments.

The author analysed PISA data to explore the relationship between students' performance and economic status, searching for differences between public and private schools across countries. He applied network-based tools to construct and cluster a bipartite network formed by countries and portions of a bidimensional space representing students’ performances and economic status. Moreover, the author conducted complementary analysis to further the understanding of network clustering. He found a cluster of countries where less privileged students perform well, being this pattern an exception to the rule.

I think the manuscript is well written, well conducted and state-of-the-art in methodology. The results are robust and the interpretations seem appropriate to the methods and results. My complete inexperience in the topic prevent me to evaluate the relevance of the results, still from a complete layman’s point of view I found the results very interesting. I did not find any point that could be further improved but this two minor suggestions:

I would like to thank the reviewer for these encouraging words. The reviewer gives an accurate summary of the work despite being, as he/she claims, a layman.

I had difficulty following Figure 1a. I think the lines represent isolines, but I am not sure. I would color or number the lines and provide a legend to facilitate understanding.

This is correct and I have clarified this in the figure caption. I however believe that colors and/or numbers would clutter the figure while not contributing significantly to the story, and therefore avoid to include them.

Please check “Figure ??” in lines 286 and 288

This is a LaTeX related typo that I now have corrected.

Journal Requirements:

1. When submitting your revision, we need you to address these additional requirements.
Please ensure that your manuscript meets PLOS ONE's style requirements, including those for file naming. The PLOS ONE style templates can be found at
https://journals.plos.org/plosone/s/file?id=wjVg/PLOSOne_formatting_sample_main_body.pdf
and
https://journals.plos.org/plosone/s/file?id=ba62/PLOSOne_formatting_sample_title_authors_affiliations.pdf

2. Please update your submission to use the PLOS LaTeX template. The template and more information on our requirements for LaTeX submissions can be found at http://journals.plos.org/plosone/s/latex.

I am already using this template.

3. Thank you for stating the following in the Acknowledgments Section of your manuscript:
(N. Eliasson and M. Oskarsson are acknowledged for advice on data retrieval. The computations were enabled by resources provided by the Swedish National Infrastructure for Computing (SNIC) at HPC2N partially funded by the Swedish Research Council through grant agreement no. 2018-05973.)
We note that you have provided funding information that is not currently declared in your Funding Statement. However, funding information should not appear in the Acknowledgments section or other areas of your manuscript. We will only publish funding information present in the Funding Statement section of the online submission form.
Please remove any funding-related text from the manuscript and let us know how you would like to update your Funding Statement. Currently, your Funding Statement reads as follows:
(The author received no specific funding for this work.)
Please include your amended statements within your cover letter; we will change the online submission form on your behalf.

The funding statement would then have to be updated to include ”Swedish Research Council grant agreement no. 2018-05973.”

4. We note that Figure 4 in your submission contain map images which may be copyrighted. All PLOS content is published under the Creative Commons Attribution License (CC BY 4.0), which means that the manuscript, images, and Supporting Information files will be freely available online, and any third party is permitted to access, download, copy, distribute, and use these materials in any way, even commercially, with proper attribution. For these reasons, we cannot publish previously copyrighted maps or satellite images created using proprietary data, such as Google software (Google Maps, Street View, and Earth). For more information, see our copyright guidelines: http://journals.plos.org/plosone/s/licenses-and-copyright.

We require you to either (1) present written permission from the copyright holder to publish these figures specifically under the CC BY 4.0 license, or (2) remove the figures from your submission:

a. You may seek permission from the original copyright holder of Figure 4 to publish the content specifically under the CC BY 4.0 license.

We recommend that you contact the original copyright holder with the Content Permission Form (http://journals.plos.org/plosone/s/file?id=7c09/content-permission-form.pdf) and the following text:
“I request permission for the open-access journal PLOS ONE to publish XXX under the Creative Commons Attribution License (CCAL) CC BY 4.0 (http://creativecommons.org/licenses/by/4.0/). Please be aware that this license allows unrestricted use and distribution, even commercially, by third parties. Please reply and provide explicit written permission to publish XXX under a CC BY license and complete the attached form.”

Please upload the completed Content Permission Form or other proof of granted permissions as an "Other" file with your submission.

In the figure caption of the copyrighted figure, please include the following text: “Reprinted from [ref] under a CC BY license, with permission from [name of publisher], original copyright [original copyright year].”

b. If you are unable to obtain permission from the original copyright holder to publish these figures under the CC BY 4.0 license or if the copyright holder’s requirements are incompatible with the CC BY 4.0 license, please either i) remove the figure or ii) supply a replacement figure that complies with the CC BY 4.0 license. Please check copyright information on all replacement

figures and update the figure caption with source information. If applicable, please specify in the figure caption text when a figure is similar but not identical to the original image and is therefore for illustrative purposes only.
The following resources for replacing copyrighted map figures may be helpful:

USGS National Map Viewer (public domain): http://viewer.nationalmap.gov/viewer/
The Gateway to Astronaut Photography of Earth (public domain): http://eol.jsc.nasa.gov/sseop/clickmap/
Maps at the CIA (public domain): https://www.cia.gov/library/publications/the-world-factbook/index.html and https://www.cia.gov/library/publications/cia-maps-publications/index.html
NASA Earth Observatory (public domain): http://earthobservatory.nasa.gov/
Landsat: http://landsat.visibleearth.nasa.gov/
USGS EROS (Earth Resources Observatory and Science (EROS) Center) (public domain): http://eros.usgs.gov/#
Natural Earth (public domain): http://www.naturalearthdata.com/

I have used the shape files from Natural Earth, and they explicitly state that there is no need to acknowledge them when using these files.

PLOS ONE: Edits requested on your submission PONE-D-21-35674R1 - [EMID:5136577f9c26d8f9]

We've checked your submission and before we can proceed, we need you to address the following issues:

1. We note that your manuscript is not formatted using one of PLOS ONE’s accepted file types. Please reattach your manuscript as one of the following file types: .doc, .docx, .rtf, or .tex (accompanied by a .pdf).
If your submission was prepared in LaTex, please submit your manuscript file in PDF format and attach your .tex file as “other.”

Attached tex file as ”other”.

2. Please note that funding information should not appear in the Acknowledgments section or Any other areas of your Manuscript. We will only publish funding information present in the Funding Statement section of the online submission form. Please remove any funding-related text from the manuscript.

Removed funding statements from the manuscript. But this has to appear in the final manuscript.

3. Thank you for stating the following financial disclosure:

"The author received no specific funding for this work."

At this time, please address the following queries:

a) Please clarify the sources of funding (financial or material support) for your study. List the grants or organizations that supported your study, including funding received from your institution.
b) State what role the funders took in the study. If the funders had no role in your study, please state: “The funders had no role in study design, data collection and analysis, decision to publish, or preparation of the manuscript.”
c) If any authors received a salary from any of your funders, please state which authors and which funders.

Please include your amended statements within your cover letter; we will change the online submission form on your behalf.

The only funding applicable to the present manuscript is the one in the following funding statement (already provided), which must appear in the final manuscript. ”The computations were

enabled by resources provided by the Swedish National Infrastructure for Computing (SNIC) at HPC2N partially funded by the Swedish Research Council through grant agreement no. 2018-05973.”

The statement provided by you, i.e. ”The funders had no role in study design, data collection and analysis, decision to publish, or preparation of the manuscript”, thus applies.
